# Experimental autoimmune encephalomyelitis (EAE) up-regulates the mitochondrial activity and manganese superoxide dismutase (MnSOD) in the mouse renal cortex

**DOI:** 10.1371/journal.pone.0196277

**Published:** 2018-04-24

**Authors:** Balamurugan Packialakshmi, Xiaoming Zhou

**Affiliations:** Department of Medicine, Uniformed Services University of the Health Sciences, Bethesda, Maryland, United States of America; University of Nebraska-Lincoln, UNITED STATES

## Abstract

Increases of the activity of mitochondrial electron transport chain generally lead to increases of production of ATP and reactive oxygen species (ROS) as by-products. MnSOD is the first line of defense against the stress induced by mitochondrial ROS. Our previous studies demonstrated that EAE progression increased Na,K-ATPase activity in the mouse kidney cortex. Since mitochondria are the major source of ATP, our present studies were sought to determine whether EAE progression increased mitochondrial activity. We found that severe EAE increased mitochondrial complex II and IV activities without significantly affecting complex I activity with corresponding increases of ROS in the isolated mitochondria and native kidney cortex. Severe EAE augmented both cytosolic and mitochondrial MnSOD protein levels and activities and decreased the specific activity of mitochondrial MnSOD when the total mitochondrial MnSOD activity was normalized to the protein level. Using HEK293 cells as a model free of interference from immune reactions, we found that activation of Na,K-ATPase by monensin for 24 hours increased complex II activity, mitochondrial ROS and MnSOD protein abundance, and decreased the specific activity of the mitochondrial MnSOD. Inhibition of Na,K-ATPase by ouabain or catalase attenuated the effects of monensin on the mitochondrial complex II activity, ROS, MnSOD protein level and specific activity. Kockdown of MnSOD by RNAi reduced the mitochondrial ability to generate ATP. In conclusion, EAE increases mitochondrial activity possibly to meet the energy demand from increased Na,K-ATPase activity. EAE increases mitochondrial MnSOD protein abundance to compensate for the loss of the specific activity of the enzyme, thus minimizing the harmful effects of ROS.

## Introduction

Multiple sclerosis (MS), an autoimmune disease, is characterized by damage and loss of neuronal cells caused by infiltration of auto-reactive immune cells into the central nervous system. Thus, a majority of studies have been focused on the immune and central nervous systems. However, the roles of other organs in the pathogenesis of MS and its animal model, experimental autoimmune encephalomyelitis (EAE), have also recently gained attention. For example, the gut could play a critical role in MS, because the impaired gut barrier leads to elevated translocation of MS-relevant microbiota across the intestinal barrier into circulation [1]. Moreover, UV exposure is associated with benefits against MS [2]. Skin increases release of soluble factors following UV exposure, leading to activation of immune suppressive pathways, protecting against the disease [2]. It has also been known that Na or high Na intake increases the production of pro-inflammatory cytokines and decreases the production of immune regulatory cytokines both *in vitro* and *in vivo* [3–7], and that high salt diets exacerbate EAE [4, 5]. The kidney regulates 90% of Na homeostasis [8, 9]. This raises an intriguing question as to whether the kidney plays a role in the pathogenesis of MS and EAE. In the kidney, Na in the blood is freely filtered into the renal tubules through glomeruli [10, 11] and then reabsorbed by the renal tubules. The amount of Na reabsorbed depends on the physiological and pathophysiological states of the body. The proximal tubules in the renal cortex reabsorb approximately 65% of the filtered Na, which makes them the primary site for Na reabsorption. Na enters the tubules mainly through the apical Na-H exchanger 3 (NHE3) and exits from the tubules exclusively through the basolateral Na,K-ATPase into the peritubular fluid and blood [8, 9, 11]. EAE is a progressive process, manifesting initially with a flaccid tail and delay in righting reflex (mild EAE) then advancing to paralysis of one or two hind limbs, or to quadriplegia (severe EAE). We recently demonstrated that under a normal salt diet, progression of EAE is associated with up-regulation of NHE3 and Na,K-ATPase [12], suggesting that progression of the disease is associated with increasing Na reabsorption by the proximal tubules.

Under the optimal condition, Na,K-ATPase expels 3 molecules of Na out of a cell in exchange for 2 molecules of K at the expense of 1 molecule of ATP [13]. ATP is primarily generated by electron transfer along the electron transport chain/respiration chain located in the inner membrane of the mitochondria. The respiratory chain is comprised of complex I to IV. The electron transfer from complex I to complex IV generates a proton gradient that drives ATP synthesis at complex V [14]. The electrons from NADH enter the electron transfer chain at complex I, whereas the electrons from succinate enter the chain at complex II [14]. While mitochondrial respiration synthesizes ATP, it also generates reactive oxygen species (ROS), primarily in the form of superoxide, due to the leakage of electrons to oxygen molecules. MnSOD is a member of the family of enzymes scavenging superoxide, using Mn or Cu/Zn as an oxidation and reduction active center. This family consists of MnSOD, Cu/ZnSOD and ECSOD. MnSOD is believed to be localized in the mitochondria, whereas Cu/ZnSOD is present in the cytosol. Some studies have shown that Cu/ZnSOD is present in the mitochondria as well. ECSOD, which uses Cu/Zn as the oxidation and reduction active center, is localized in the extracellular compartment [15]. The accumulation of superoxide in the mitochondria is harmful to cells. As the first line of defense against mitochondrial superoxide, MnSOD converts superoxide into hydrogen peroxide, which either acts as a signaling molecule and/or is then detoxified by other antioxidant enzymes such as catalase and peroxidases [15, 16].

Since progression of EAE increases Na,K-ATPase activity [12], and because the increase of Na,K-ATPase activity stimulates cell respiration [17], in the present studies, we examined whether EAE increased the mitochondrial activity in the renal cortex. We found that EAE progression increased the activities of mitochondrial complex II and IV. EAE increases production of a wide variety of pro- and anti-inflammatory cytokines, which have diversified effects on the mitochondrial function [18–20]. To explore whether stimulation of Na,K-ATPase was responsible, at least in part, for EAE-induced increase of the mitochondrial function in the kidney, we then examined the effect of monensin, a Na,K-ATPase activator, on the activity of the mitochondrial electron transport chain in HEK293 cells, which are free of any immunological responses. We uncovered that activation of Na,K-ATPase alone was sufficient to increase the mitochondrial complex II activity.

## Materials and methods

### Induction of EAE

EAE was induced as previously described [12]. All procedures were approved by Uniformed Services University Institutional Animal Care and Use Committee. Briefly, each male C57BL/6J (The Jackson Laboratory, 9 to 16 weeks old) was injected subcutaneously with PBS (Control) or 100 μg MOG_35-55_ (New England Peptide) emulsified in 100 μl complete Freund’s adjuvant containing 300 μg Mycobacterium tuberculosis (Fisher Scientific) and 100 ng pertussis toxin (List Biological Laboratories) intraperitoneally. The same dose of pertussis toxin was given two days later via the same route to boost the immune reaction. The mice were sacrificed 24–48 hours after they manifested mild (score 1 and 2) or severe (score 3 and 4) EAE symptoms by CO_2_ inhalation followed by cervical dislocation. To facilitate sick mice to access food and water, water-replete gel food and regular pellet chow were supplemented on the floor of cages. The sickness was scored as 1-Flaccid Tail, 2-Delay in righting reflex, hind limb weakness, 3-Flaccid paralysis in 1 hind limb, 4-Flaccid paralysis in both hind limbs and 5-Quadriplegia or Moribund [21].

### Water and food restrictions

Water and food restrictions were performed as previously reported [12]. Briefly, in the water restriction study, mice were placed in metabolic cages (Hatteras Instruments) and adapted to a water replete gel diet containing 4 g powder food (OpenSource Diet), 0.09 g agar and 5 ml water/20g body weight/24 hours for 48 hours. Then control mice were fed with the same diet and same ration for 28 hours. Water-restricted mice were rationed with 4 g powder food, 0.05 g agar and 1 ml water/20g body weight/24 hours for 28 hours. In the food restriction study, control mice were treated same as in the water restriction study, whereas the food and water supplies were reduced by 80% for 48 hours in the food-restricted group. Mice were sacrificed immediately after the treatments.

### Western analysis

The renal cortex was homogenized in 10 mM triethanolamine, pH 7.4 and 250 mM sucrose plus a protease inhibitor tablet (Roche) and phosphatase inhibitors 2.0 mM NaF and 2.0 mM Na_3_VO_4_. The homogenates were centrifuged at 13000 rpm at 4°C for 8 min. The protein concentrations were measured with BCA assay (ThermoFisher). A brief sonication was employed to break down HEK293 cells mitochondrial DNA. The samples (30 μg/lane for the kidney cortex extracts and 10 μg/lane for HEK293 cell extracts in most cases) were fractionated in 4–12% Bis-Tris gels (Invitrogen). The antibodies against NHE3 (sc-16103), Na,K-ATPase α1 (sc-21012), COX-4 (sc-69360), aldose reductase (sc-17736) and actin (sc-1615) were purchased from Santa Cruz Biotechnology. The mouse anti MnSOD (MAB4081, Millipore) was used to probe MnSOD from the kidney cortex, whereas the rabbit anti MnSOD (06–984, EMD) was used to hybridize MnSOD from HEK293 cells. The antibody against CuZnSOD (07–403) was purchased from Upstate Biotechnology. The antibodies against GAPDH (2118) and Bcl-2 (2876) were purchased from Cell Signaling Technologies.

### qPCR

Total RNA was extracted with the ice-cold RNAzol RT kit (Molecular Research Center) and quantified with NanoDrop (ThermoFisher). cDNAs were synthesized with the High Capacity cDNA Reverse Transcription Kit (Applied Biosystems). mRNAs were quantified with a SYBR Green PCR kit (QuantiFast, Qiagen) in Stratagene Mx3005P (Agilent Technologies). The primers for MnSOD are 5’-GTGGAGAACCCAAAGGAGAG-3’ (forward) and 5’-AACCTTGGACTCCCACAGAC-3’ (reverse). The primer for Bcl-2 are 5’-ACTTCTCTCGTCGCTACCGT-3’ (forward) and 5’-TCCCTGAAGAGTTCCTCCAC-3’ (reverse). The amount of 26.8 ng total RNA/reaction was used. mRNA abundance was not normalized to 18s rRNA. Fold difference in mRNA abundance between conditions (*F*) was calculated as described previously [22].

### MnSOD activity assay

The MnSOD activity was measured with the SOD Assay Kit (Sigma 19160) based on the manufacturer’s recommendation. 15 μg of protein extracts from each fraction was used with the same amount of proteins that was boiled for 8 minutes as a blank control. The samples were pre-incubated with 100 mM NaCN at room temperature for 10 minutes to inhibit CuZnSOD. Absorption was recorded at 450 nm. The total MnSOD activity is defined by the difference in A_450_ between the blank control and with protein extracts. The specific MnSOD activity is the total activity normalized by the MnSOD protein abundance in each sample.

### Mitochondrial ability to generate ROS

Mitochondrial ability to generate ROS was measured as previously described [23]. Briefly, mitochondrial extracts from the renal cortex or HEK293 cells (5 μg/assay) were incubated in 50 mM K_3_PO_4_ pH 7.8, 1 mM DETAPAC, 1 unit catalase, 0.5 mg/ml salmon sperm DNA, 80 μM antimycin A, 5 mM succinate, 10 μM dihydroethidium in a black viewplate (PerkinElmer) at 37^0^ C for 30 min. Fluorescence was measured with excitation at 485 nm and emission at 610 nm.

### Imaging ROS in the renal cortex

Dihydroethidium was dissolved in DMSO and injected intraperitoneally at 50 mg/kg. Mice were sacrificed 18 hours after injection and perfused retro-ventrically first with ice-cold PBS and then with 10% formalin. The kidney was frozen sectioned at 30 μm/slice and imaged with Zeiss 710 40X oil lens at excitation 568 nm and emission 605 nm [24].

### Cells and treatments

HEK293 cells were purchased from ATCC and cultured at 37^0^ C in DMEM (Sigma, D6429) plus 10% of fetal bovine serum. The cells were used between passage 41 and 48. Results were not consistent when the cells were used beyond passage 48. With the exception of flow cytometry analysis, the cells were pre-incubated with 400 U/ml catalase for 30 minutes or 4 nM ouabain for 60 minutes before monensin was added.

### Transfection of siRNAs

The flexi tube siRNAs against human MnSOD (GS6648) and Na,K-ATPase α1-subunit (GS476) were purchased from Qiagen and transfected into HEK293 cells with Lipofectamine 2000 (ThermoFisher) or with HiPerFect (Qiagen) according to the manufacturers’ protocols. The control siRNA was same as before [25].

### Flow cytometry analysis

HEK293 cells were placed down at 7X10^5^ cells/well in a 6-well plate for 20 to 24 hours. Next day, the cells were treated with ouabain (4 nM) for 60 min, then with 0.1% ethanol (control for monensin) or 10 μM monensin in the presence of ouabain for additional 60 min, before the cells were loaded with 2.5 μM Mito-sox Red (ThermoFisher) for another 60 min. The cells were collected with trypsinization and analyzed with LSRII flow cytometer (Becton Dickinson) with excitation at 480 nm and emission at 570 nm.

### Isolation of mitochondria

Mitochondria from the renal cortex and HEK293 cells were isolated as previously described with slight modifications [26]. Briefly, tissue or cells were homogenized in IBcell buffer with a glass homogenizer driven by an electrically powered motor (Wheaton Overhead Stirrer). The homogenates were centrifuged at 4°C at 600 g for 20 minutes. The supernatants were collected and centrifuged at 4°C at 10,000 g for 10 minutes. The supernatants (the cytosolic fraction) were collected, and the pellets (the mitochondrial fraction) were washed once with the same buffer and centrifuged at 4°C at 10,000 g for 10 minutes to pellet mitochondria. The protein concentrations in each fraction were measured by BCA assay.

### Mitochondrial function and ATP level assays

The activities of the mitochondrial complex I and complex II were measured as previously described [27]. Briefly, for measuring complex I activity, mitochondrial extracts (5 μg) were pre-incubated with 25 mM potassium phosphate, 3.5 g/L BSA, 60 μM 2,6-dichloroindophenol, 70 μM decylubiquinone, 1 μM antimycine A at 37^0^ C for 3 min. The complex I activity was activated by adding 0.2 mM NADH, then rotenone (1 μM) was added to generate superoxide, which oxidizes 2,6-dichloroindophenol, resulting in decreases at A_630_. The difference in A_630_ with and without mitochondria reflects the activity of complex I. For measuring complex II activity, mitochondrial extracts (5 μg) were pre-incubated with 80 mM potassium phosphate, 1 g/L BSA, 2 mM EDTA, 0.2 mM ATP, 80 μM 2,6-dichloroindophenol, 50 μM decylubiquinone, 1 μM antimycine A and 1 μM rotenone at 37^0^ C for 10 min. The complex II activity was activated and superoxide was generated by adding 10 mM succinate and 2 mM KCN. Complex IV activity was measured as previously described [28]. Briefly, mitochondrial extracts (5 μg) were incubated with 10 mM potassium phosphate, 1 g/L BSA, 50 μM reduced cytochrome c (Abcam), 0.3 M sucrose and 10 mM laurylmaltoside at 37^0^ C for 12 min. The increases in A_562_ indicate the increases in complex IV activity.

The cellular ATP levels were measured with a luminescence-based ATP Determination Kit (Molecular Probes, A22066) according to the manufacturer’s protocol. The luminescence signals generated by luciferase are proportional to the cellular ATP levels.

### Statistical analysis

In the kidney cortex analyses, all results were normalized to the results from the first mouse in the control group except for the mitochondrial activity assays in which the differences in spectrophotometric readings were presented. In the cell culture studies, results were normalized to the control in each individual experiment except for the mitochondrial function assays in which the differences in spectrophotometric readings were presented and mitochondrial ROS assays in which mean fluorescence intensity (MFI) was presented. Data are expressed as mean ± SEM. Statistical analyses were performed by non-paired *t* test, paired *t* test, One-way ANOVA and Two-way ANOVA with Turkey’s multiple comparisons as appropriate. P ≤0.05 is considered significant.

## Results

### EAE increases the mitochondrial activity in the renal cortex

**Fig 1:**
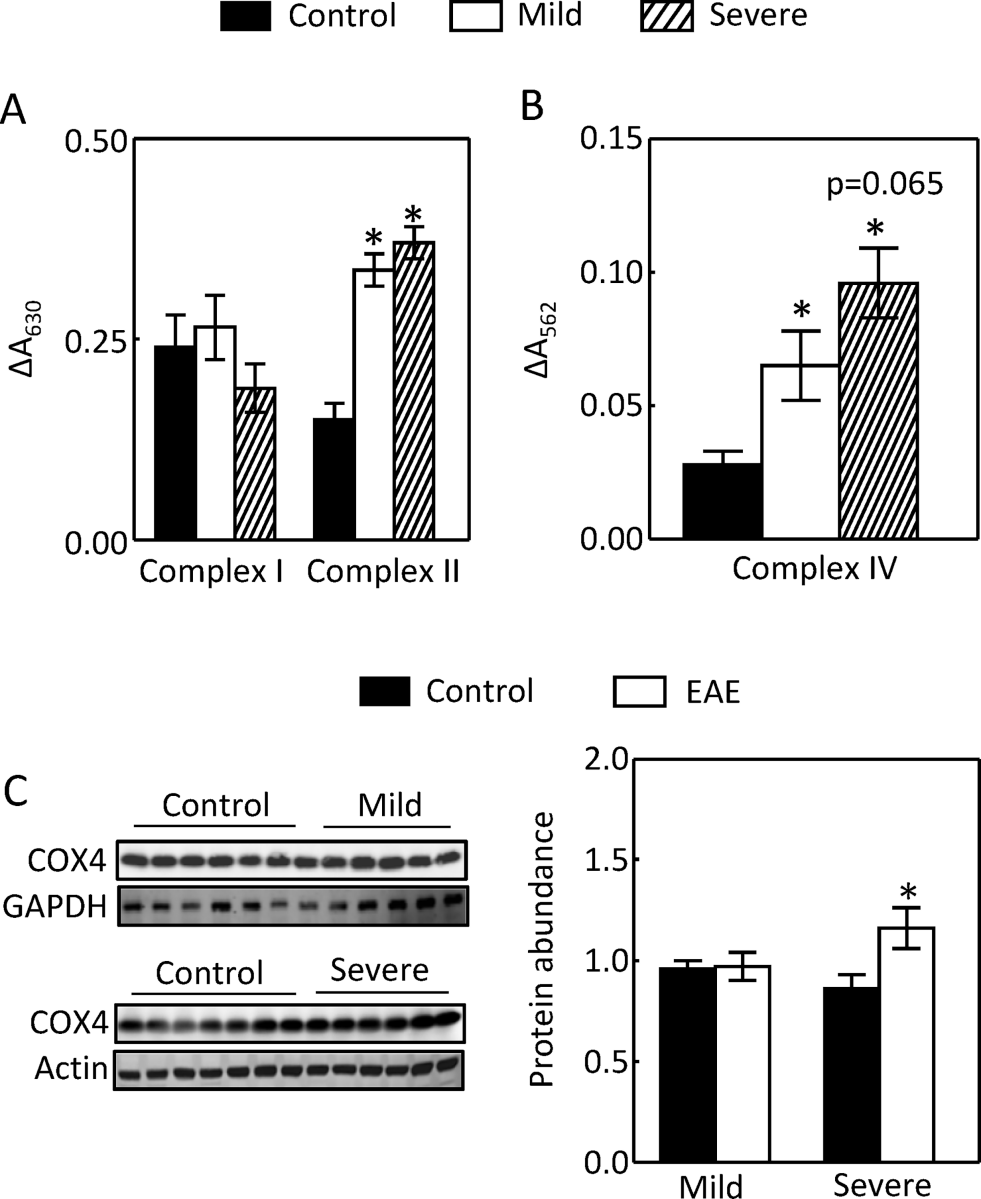
EAE increased the activities of mitochondrial complex II and IV, but not complex I from the mouse renal cortex (A and B). Severe EAE had a trajectory to further augment the complex IV activity over mild EAE. This effect was associated with an increase of COX4 protein level (B and C). The activities of complex I and II were measured by the difference of absorption at A_630_ between the assay buffer alone and assay buffer with 5 μg mitochondria extracts (A). The complex IV activity was assayed by increases in absorption at A_562_ in the presence of 5 μg mitochondria extracts (B). The protein extracts from mouse kidney cortex were separated in a 4–12% Bis-Tris gel (Invitrogen) and probed with antibodies (C). (*p < 0.05 vs Control, p = 0.065 vs mild EAE; Control n = 7, Mild EAE n = 5, Severe EAE n = 6; One-way ANOVA for A and B, unpaired *t* test for C).

We found that both mild (scores 1 and 2) and severe (scores 3 to 5) EAE increased the mitochondrial complex II and IV activities, but not the complex I activity (Fig 1A and 1B). Further, severe EAE elevated the protein level of cytochrome c oxidase subunit IV (COX4), a critical component of complex IV, by 35% (Fig 1C).

### Severe EAE increases ROS

**Fig 2:**
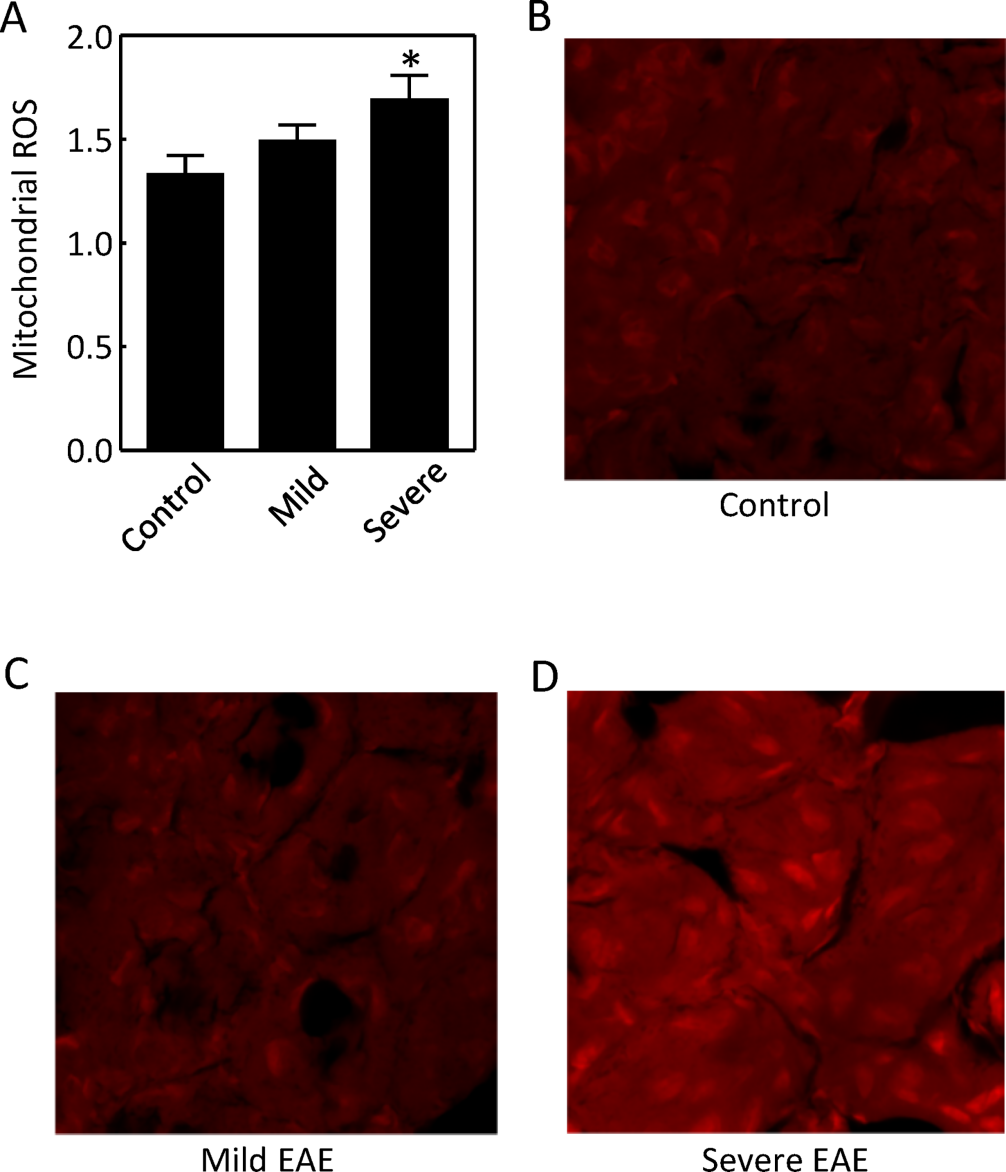
Severe EAE increased production of ROS from the isolated mitochondria in the mouse renal cortex (A) and in the native renal cortex (D). Mitochondrial ROS production was measured by increases of fluorescence from dihydroethidium (A). The same dye (50 mg/kg) was injected intraperitoneally. The frozen sections of the kidney cortex were imaged 18 hours after injection, representatives of two independent experiments (B to D) (*p < 0.05 vs Control, One-way ANOVA, the sample sizes are same to those in Fig 1).

Since elevation of the mitochondrial function increases the mitochondrial ROS, as an additional test of the effect of EAE on the mitochondrial function, we first isolated mitochondria from the kidney cortex and examined their ability to generate ROS with the fluorescent dye dihydroethidium in a reaction buffer. Severe EAE enhanced mitochondrial ability to generate ROS (Fig 2A). We then injected the fluorescent dye dihydroethidium into mice for 18 hours and directly imaged ROS in frozen sections of the cortical slices. Severe EAE elevated ROS in the kidney cortex (Fig 2B to 2D).

### Severe EAE increases protein abundance of MnSOD, but not of CuZnSOD in the kidney cortex

**Fig 3:**
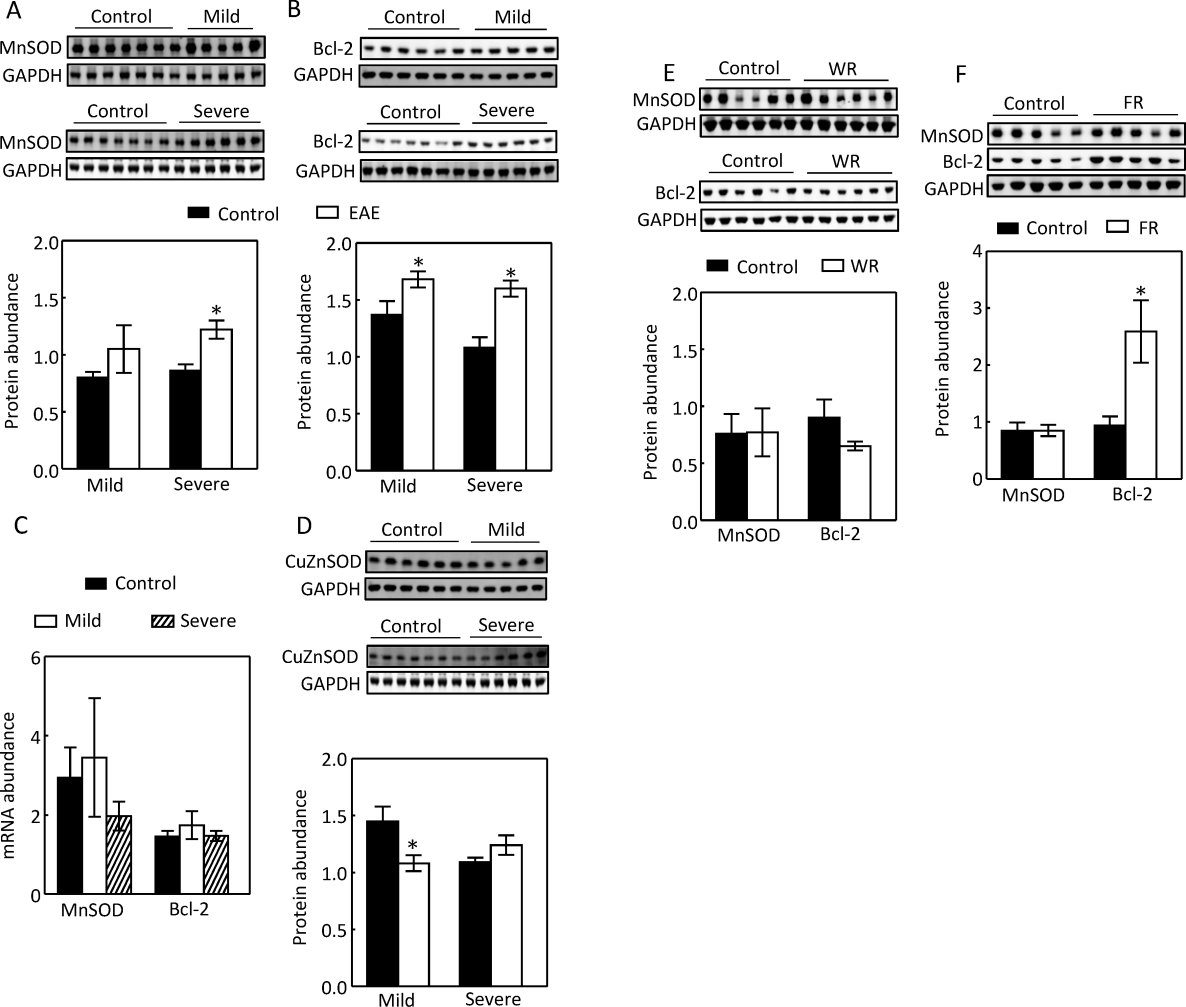
Severe EAE increased the total MnSOD protein abundance (A) and had no significant effect on CuZnSOD protein level (D) in the kidney cortex. Both mild and severe EAE elevated the protein level of Bcl-2 (B). EAE had no significant effect on the mRNA level of either MnSOD or Bcl-2 (C). Water restriction (WR) had no significant effect on either the total MnSOD or Bcl-2 protein level (E). Food restriction (FR) had no significant effect on the total MnSOD protein abundance, but increased the total Bcl-2 protein level (F). Protein levels were analyzed as in Fig 1C. The total RNA was extracted with RNAzol RT kit (Molecular Research Center), and MnSOD and Bcl-2 mRNAs were quantified with SYBR-based qPCR. (*p < 0.05 vs Control, unpaired *t* test).

Severe EAE increased protein abundance of the total MnSOD by 44% (Fig 3A). Besides MnSOD, Bcl-2 also plays a critical role in protecting mitochondria from oxidative injury [29]. Both mild and severe EAE increased protein abundance of Bcl-2 by 23 and 48%, respectively, (Fig 3B). These effects were apparently post-transcriptional, since the mRNA level of MnSOD or Bcl-2 was not significantly altered under either mild or severe EAE (Fig 3C). In contrast, EAE progression did not elevate the protein level of CuZnSOD (Fig 3D). It is not clear why mild EAE decreased the protein abundance of CuZnSOD (Fig 3D). Since the mice with severe EAE were dehydrated [12], we restricted mice water intake for 28 hours [12] and found that water restriction had no significant effect on the protein abundance of MnSOD or Bcl-2 (Fig 3E). EAE induces inflammatory anorexia [30], as such, we reduced supplies of food and water by 80% for 48 hours [12]. However, food restriction had no significant effect on MnSOD protein, but increased Bcl-2 protein (Fig 3F). Therefore, we conclude that EAE progression specifically elevates the protein level of MnSOD. These results are consistent with the observation that EAE increased the activity of mitochondria.

### EAE progression up-regulates both cytosolic and mitochondrial MnSOD

**Fig 4:**
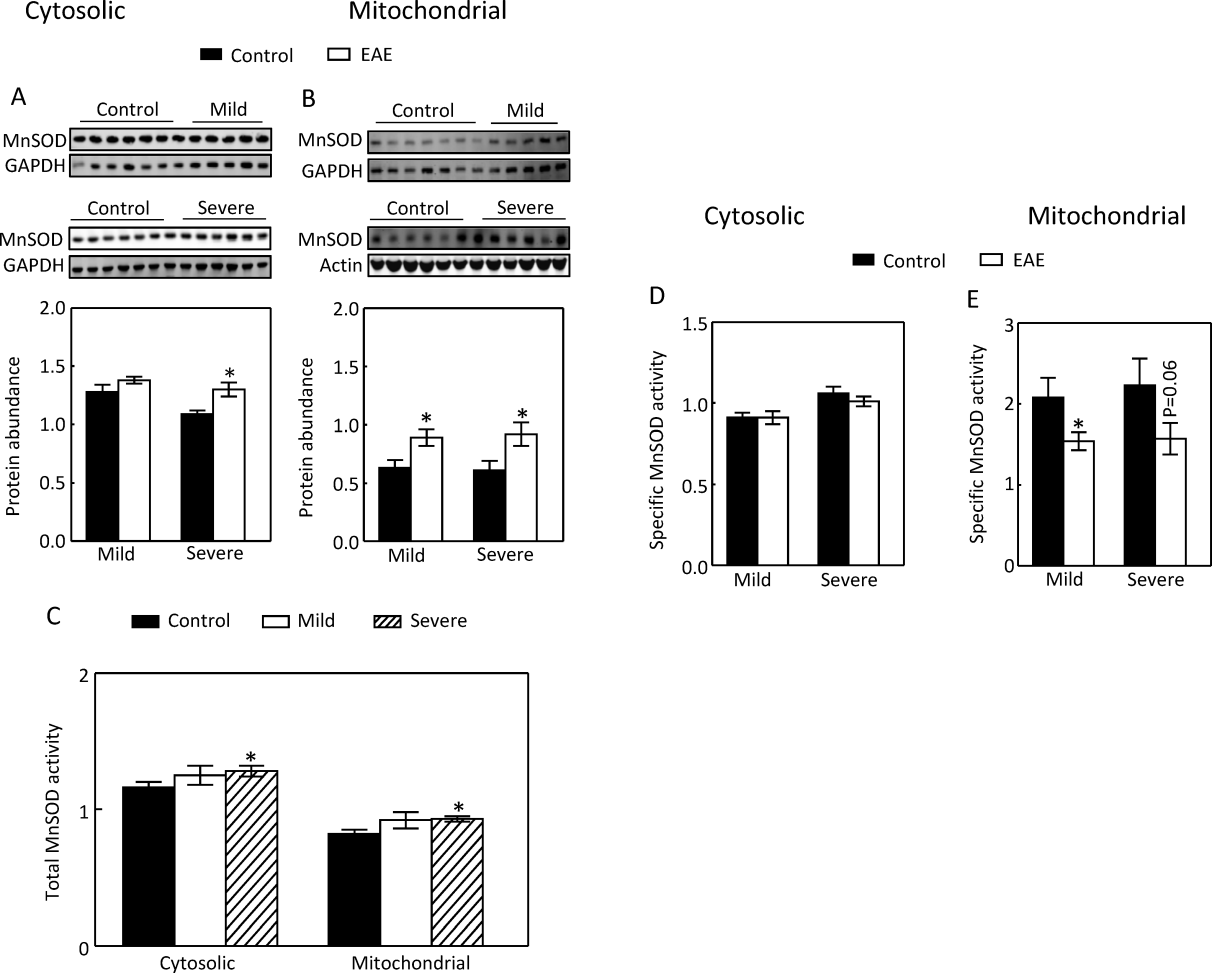
Severe EAE increased the cytosolic MnSOD protein abundance (A). Both mild and severe EAE elevated the mitochondrial MnSOD protein levels (B). Severe EAE increased the total activities of cytosolic and mitochondrial MnSOD (C). EAE had no significant effect on the specific activity of cytosolic MnSOD (D), but reduced the specific activity of mitochondrial MnSOD (E). The cytosolic and mitochondrial proteins were separated by centrifugation and analyzed by Western analysis as in Fig 1C. Total MnSOD activity was measured by the difference of absorption at A_450_ between heat-inactivated and live extracts. The readings were normalized to the reading from the first mouse in the control group. The specific MnSOD activity is expressed by the total MnSOD activity normalized to MnSOD protein abundance. (*p < 0.05 vs Control, unpaired *t* test except for C by One-way ANOVA).

Although MnSOD is believed to be in mitochondria [15], one study reports that it is also present in the cytosol of mammalian cells [31]. To determine whether MnSOD was also present in the cytosol of the renal cortex, and MnSOD in which compartment EAE affected, we separated the mitochondrial proteins from the cytosolic ones and analyzed them with Western analysis. Severe EAE significantly increased both cytosolic and mitochondrial MnSOD protein abundance, but mild EAE only elevated the mitochondrial MnSOD protein abundance, not the cytosolic one (Fig 4A & 4B). Severe EAE modestly, but significantly, increased the total MnSOD activity in both the cytosol and the mitochondria (Fig 4C). However, after normalizing the total MnSOD activity with MnSOD protein, we found that although EAE did not significantly affect the specific MnSOD activity in the cytosol (Fig 4D), EAE actually reduced the specific MnSOD activity in the mitochondria (Fig 4E). We conclude that EAE progression increases the cytosolic and mitochondrial MnSOD protein levels accompanied with marginal increases of the total MnSOD activity in both compartments, and that the effect of severe EAE on the mitochondrial MnSOD protein level is compensatory for the loss of the specific activity of mitochondrial MnSOD to minimize the deleterious effect of the mitochondrial superoxide.

### Activation of Na,K-ATPase increases the activity of mitochondrial complex II and cytosolic ATP in HEK293 cells

**Fig 5:**
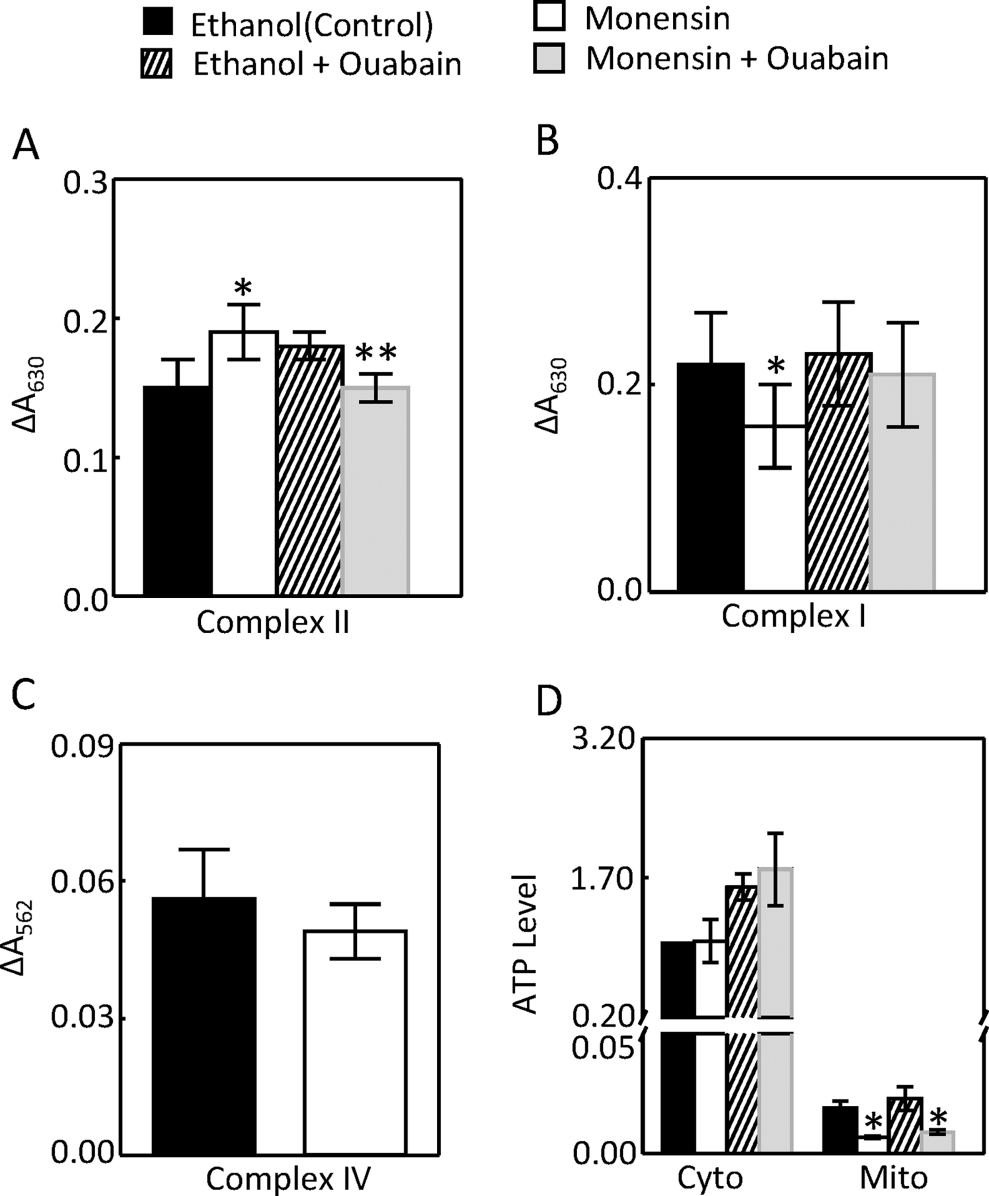
Monensin (10 μM) increased the mitochondrial complex II activity (A, n = 5), but decreased the complex I activity (B, n = 5) and had no significant effect on the complex IV activity (C, n = 4). Ouabain (4 nM) blocked the effect of monensin on the complex II activity (A). Monensin had no significant effect on the cytosolic ATP level, but decreased the mitochondrial ATP level (D, n = 6). HEK293 cells were pre-incubated with ouabain for 60 min and then monensin was added for 24 hours before they were collected. The complex I, II and IV activities were measured as in Fig 1A and 1B. ATP level was measured by a luminescence-based ATP Determination Kit from Molecular Probes. (*p < 0.05 vs Control, **p < 0.05 vs Monensin Two-way ANOVA).

The effect of severe EAE on mitochondrial function could be due to the demand for more ATP resulted from increased Na,K-ATPase activity [12] or from immunological reactions independent of Na,K-ATPase [18–20]. The ionophore monensin increases Na transport by catalyzing Na-H exchange, increasing the intracellular Na concentration, and leading to activation of Na,K-ATPase to expel Na from cells [32]. Monensin also stresses the endoplasmic reticulum [33]. To determine whether monensin could recapitulate the effect of EAE on the mitochondrial function and whether its effect was mediated through activation of Na,K-ATPase, we examined the effect of monensin on the activities of complex I, II and IV, and whether these effects were blunted by inhibition of Na,K-ATPase. We chose to use 10 μM monensin, because monensin at this dose produced reliable data in measurements of the mitochondrial complex II, ROS and MnSOD activities. To determine whether the effects of monensin were dependent on Na,K-ATPase, we initially used siRNAs against the Na,K-ATPase α1-subunit. However, the siRNAs did not reduce Na,K-ATPase activity, although it knocked down the α1-subunit protein (data not shown). This paradoxical effect could be due to the compensatory effect from other isoforms of the enzyme. We then found that ouabain, a specific inhibitor of all isoforms of Na,K-ATPase, at 4 nM inhibited the Na,K-ATPase activity without causing notable cell death (data not shown). Similarly like progression of EAE, monensin increased the activity of mitochondrial complex II, and this effect was blocked by 4 nM ouabain (Fig 5A). Monensin significantly reduced the complex I activity and had no significant effect on the complex IV activity (Fig 5B & 5C). Monensin had no significant effect on the cytosolic ATP level, but significantly decreased the mitochondrial ATP level. Ouabain had a trajectory to increase the cytosolic ATP level, but the effect did not reach statistical significance, probably because a significant portion of Na,K-ATPase still consumed ATP when ouabain was used at 4 nM (Fig 5D). We conclude that the activation of Na,K-ATPase alone is sufficient to stimulate the mitochondrial activity. We could not detect ATP from the mouse kidney cortex. This was because mice did not simultaneously develop EAE, and in order to process all kidneys at the same time, some mouse kidneys had to be stored at -80°C. However, ATP was degraded during storage.

### Activation of Na,K-ATPase increases the mitochondrial ROS

**Fig 6:**
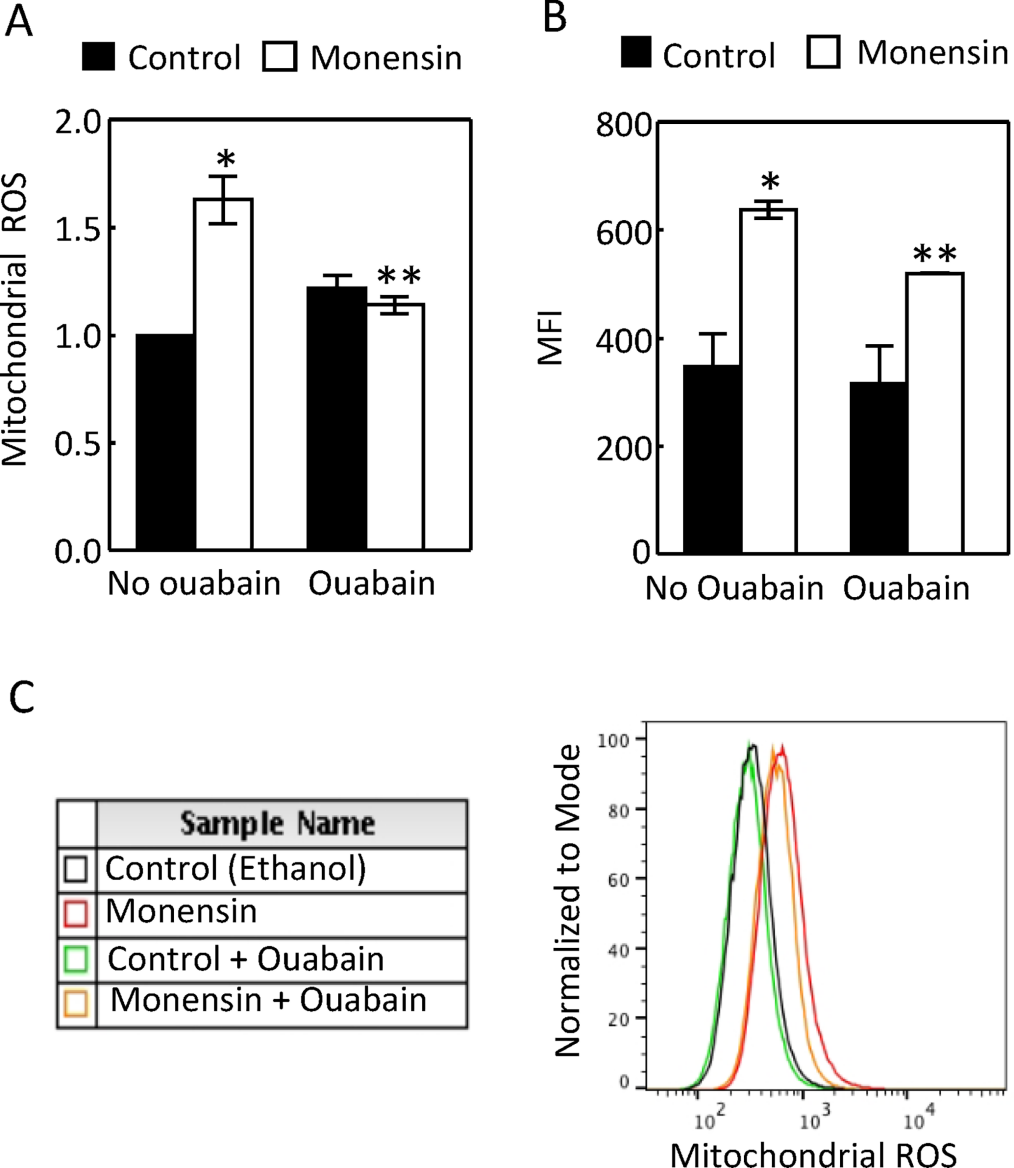
Monensin increased the mitochondrial ROS in the isolated mitochondria (A, n = 5) and in live HEK293 cells (B, n = 4 and C). Ouabain inhibited the effect of monensin. (A) HEK293 cells were treated as in Fig 5 and ROS from isolated mitochondria were measured as in Fig 2A. B is average of 4 independent flow cytometry analyses of mitochondrial ROS in live HEK293 cells. MFI: mean fluorescence intensity. C is a representative flow cytometry analysis. After HEK293 cells were incubated with 4 nM ouabain for 60 min, monensin (10 μM) was added into the medium and the cells were incubated for an additional 60 min, then Mito-sox Red (2.5 μM) was added and the cells were incubated for another 60 min before they were collected for analysis. (*p < 0.05 vs Control, **p < 0.05 vs Monensin, Two-way ANOVA).

To directly test whether stimulation of Na,K-ATPase increased mitochondrial ROS, we first treated HEK293 cells with monensin with or without ouabain for 24 hours and measured ROS from the isolated mitochondria and found that monensin increased mitochondria ability to generate ROS, and this effect was abolished by ouabain (Fig 6A). We then measured the mitochondrial ROS with MitoSox Red, a specific probe for the mitochondrial ROS, in live HEK293 cells with flow cytometry. Monensin increased the mitochondrial ROS, and ouabain attenuated this effect (Fig 6B & 6C). These data are consistent with the conclusion that activation of Na,K-ATPase stimulates mitochondrial activity.

### Activation of Na,K-ATPase elevates the mitochondrial MnSOD protein level

**Fig 7:**
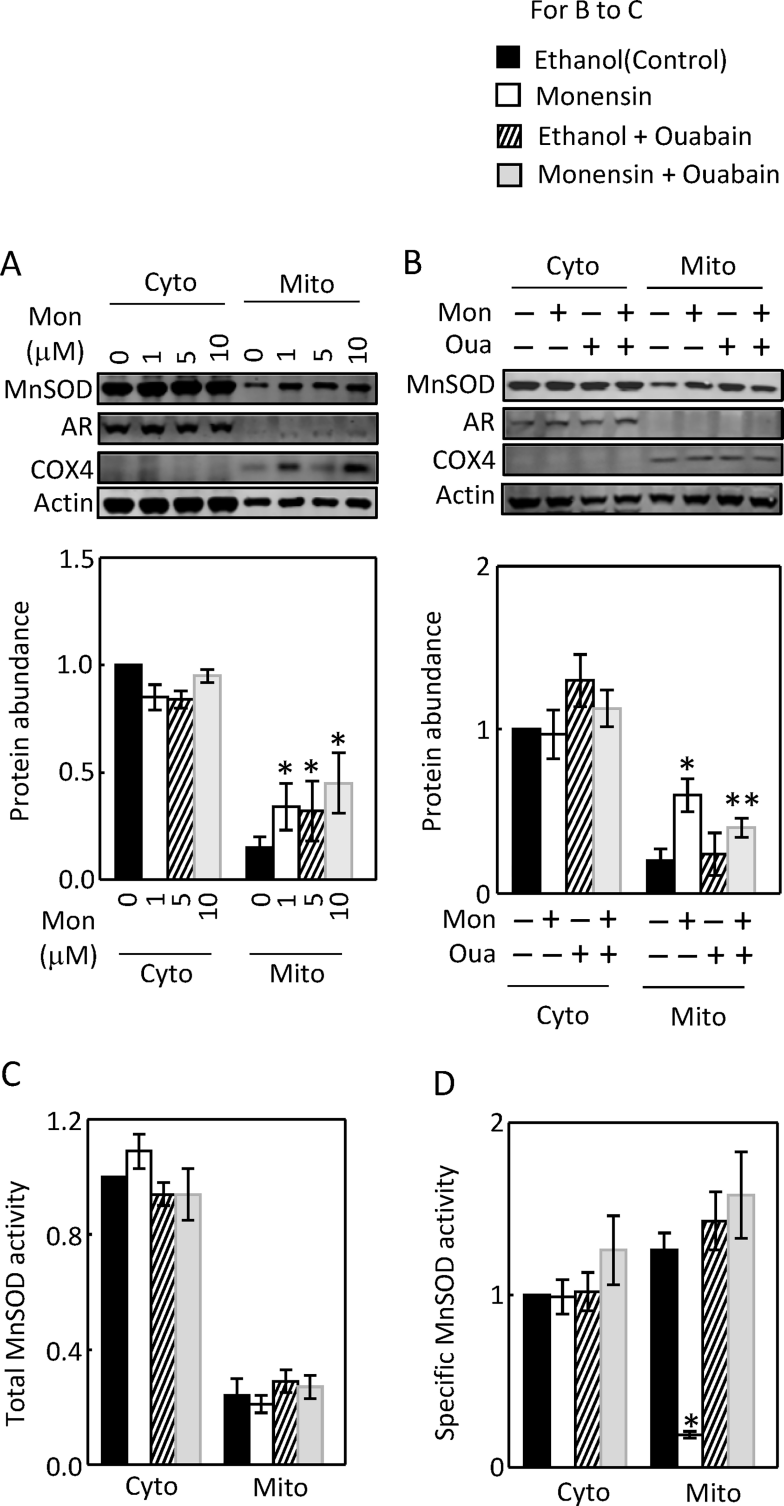
Monensin elevated the mitochondrial MnSOD protein level without a significant effect on the cytosolic MnSOD protein abundance (A, n = 5). Ouabain (4 nM) inhibited the effect of monensin on the mitochondrial MnSOD protein (B, n = 5). Membranes were probed with antibodies against aldose reductase (AR, cytosolic marker) and cytochrome c oxidase subunit IV (COX4, mitochondrial marker) to show adequate separation of these two compartments and with actin antibody to show roughly equal loading. Monensin had no significant effect on the total activity of cytosolic or mitochondrial MnSOD (C, n = 5), but significantly reduced the specific activity of mitochondrial MnSOD and this effect was blocked by ouabain (D, n = 5). HEK293 cells were treated as in Fig 5. The total and specific MnSOD activities were measured as in Fig 4, but the total and specific activities were normalized to the control in each experiment. (*p < 0.05 vs Control or Monensin plus Ouabain in D, **p < 0.05 vs Monensin, One-way ANOVA for A, Two-way ANOVA for B and D).

Monensin increased the mitochondrial MnSOD protein level as low as 1 μM, but not the cytosolic one even at 10 μM (Fig 7A). The effect of monensin on the mitochondrial MnSOD was inhibited by 4 nM ouabain (Fig 7B). Monensin had no significant effect on the total MnSOD activity in either compartment (Fig 7C). However, monensin reduced the specific activity of mitochondrial MnSOD. Ouabain blocked this effect (Fig 7D). We conclude that similarly like the effect of severe EAE, activation of Na,K-ATPase elevates the mitochondrial MnSOD protein level to compensate for ROS-induced loss of the MnSOD specific activity.

### Catalase abrogates monensin-induced mitochondrial ROS

**Fig 8:**
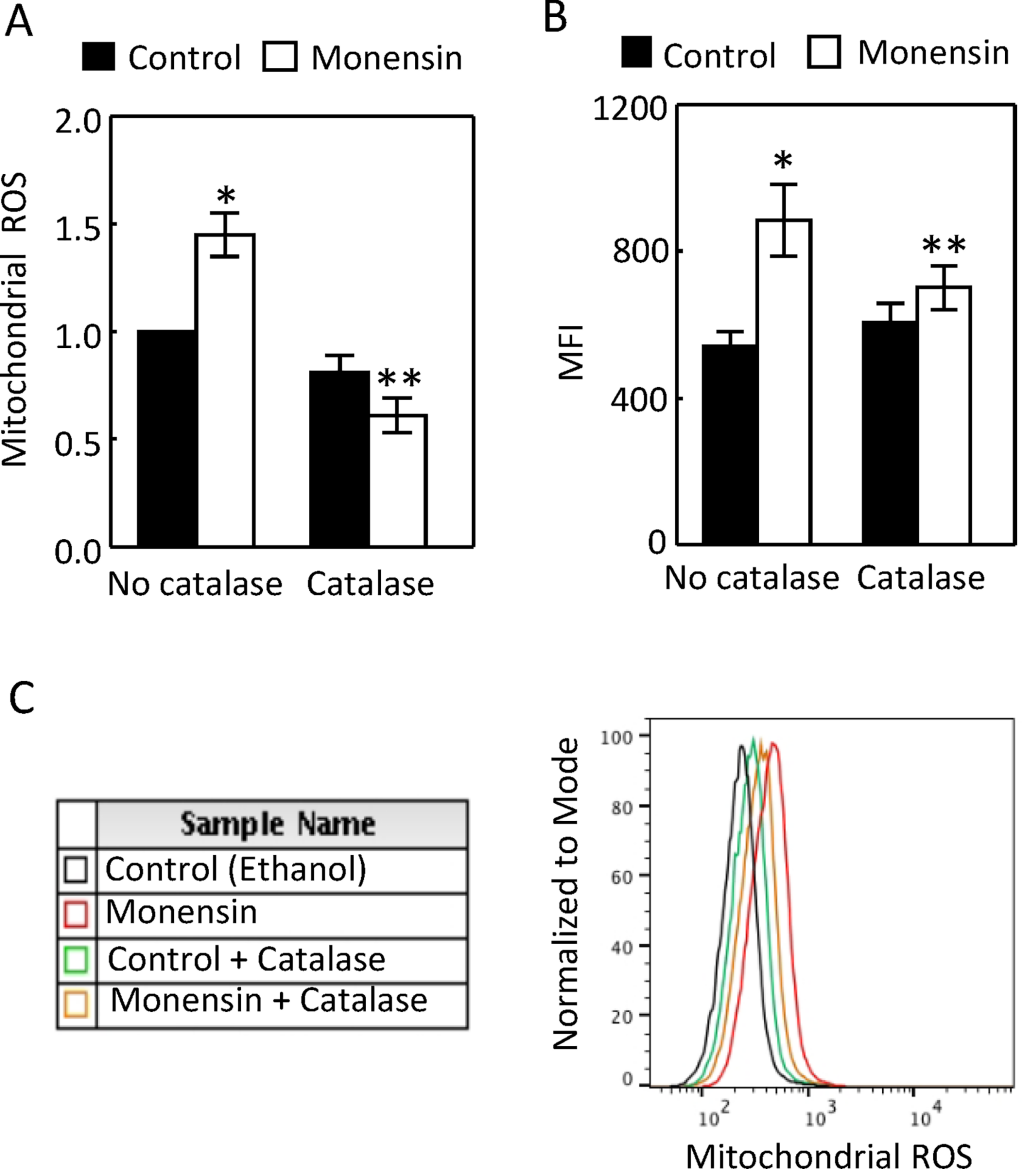
Catalase reduced the monensin-induced increases of mitochondrial ROS in the isolated mitochondria (A) and in live HEK293 cells (B, n = 5 and C, n = 5). Mitochondrial ROS were measured as in Fig 6 except ouabain was replaced by catalase (400U/ml) (*p < 0.05 vs Control, **p < 0.05 vs Monensin, Two-way ANOVA).

We first tested whether atpenin, a specific inhibitor of the complex II [34], reduced monensin-triggered mitochondrial ROS, but we found that atpenin actually increased the mitochondrial ROS by flow cytometry (data not shown). We then treated HEK293 cells with 400 U/ml catalase. Catalase reduced the mitochondrial ROS with the measurements either in the isolated mitochondria or in live cells (Fig 8).

### ROS contributes to the monensin-induced up-regulation of Na,K-ATPase

**Fig 9:**
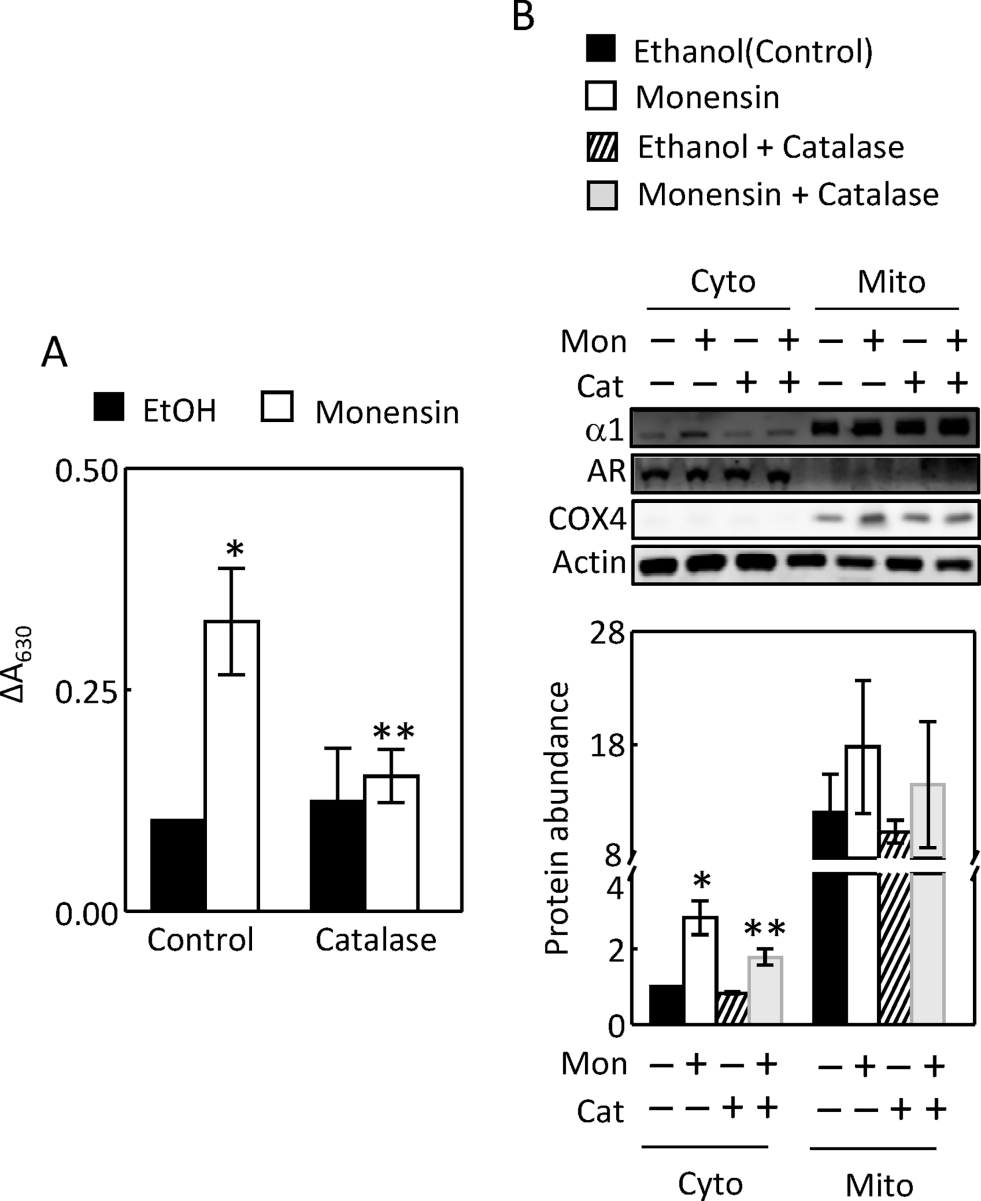
Catalase reduced the monensin-induced increases of Na,K-ATPase activity (A, n = 4) and the cytosolic Na,K-ATPase α1-subunit protein abundance (B, n = 4). The Na,K-ATPase activity was measured by ouabain-sensitive release of inorganic phosphate, which reacted with malachite green and was quantified by increases in the absorption at A_630_. Membranes were probed with antibodies against aldose reductase (AR, cytosolic marker) and cytochrome c oxidase subunit IV (COX4, mitochondrial marker) to show adequate separation of these two compartments and with actin antibody to show roughly equal loading. (*p < 0.05 vs Control, **p < 0.05 vs Monensin, Two-way ANOVA, n = 4).

We previously demonstrated that ROS contributes to low K-induced up-regulation of Na,K-ATPase in MDCK cell [35]. We found that catalase reduced the monensin-induced increases of Na,K-ATPase activity (Fig 9A) and cytosolic α1-subunit protein level (Fig 9B), indicating that ROS also contributes to monensin-induced up-regulation of Na,K-ATPase.

### Catalase decreases the monensin-induced increases of the complex II activity

**Fig 10:**
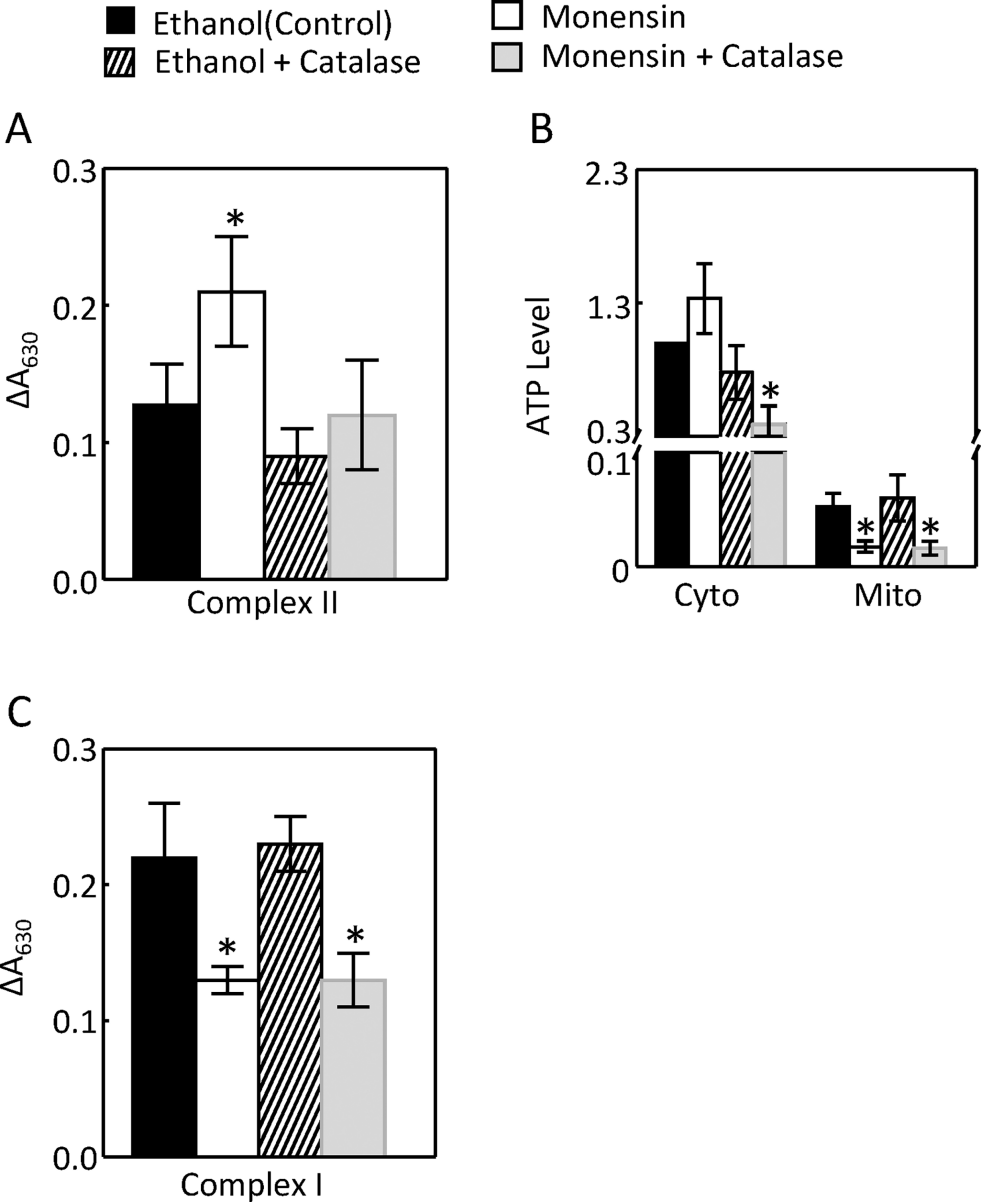
Catalase reduced the monensin-induced increases of complex II activity (A, n = 5) and cytosolic ATP level in the presence of monensin (B, n = 6). Monensin reduced the mitochondrial ATP level and complex I activity, and catalase did not significantly affect the effects of monensin (B and C, n = 4). The complex I and II activities and ATP levels were measured as in Fig 5 except ouabain was replaced by catalase (400 U/ml). (*p < 0.05 vs Control, **p < 0.05 vs monensin, Two-way ANOVA).

Since ouabain inhibited the monensin-induced increase of complex II activity (Fig 5A), and since catalase reduced the monensin-induced up-regulation of Na,K-ATPase (Fig 9), we examined the effect of catalase on the complex II activity and ATP level. Catalase inhibited the monensin-induced increase of complex II activity (Fig 10A). Again, monensin had no significant effect on the cytosolic ATP level, but significantly decreased the mitochondrial ATP level. It is not clear why catalase reduced the cytosolic ATP level in the presence of monensin (Fig 10B). Monensin reduced the complex I activity, and catalase did not affect the effect of monensin (Fig 10C). These data provide an additional piece of evidence supporting that the activation of Na,K-ATPase increases complex II activity.

### ROS contributes to the monensin-induced increase of mitochondrial MnSOD protein abundance

**Fig 11:**
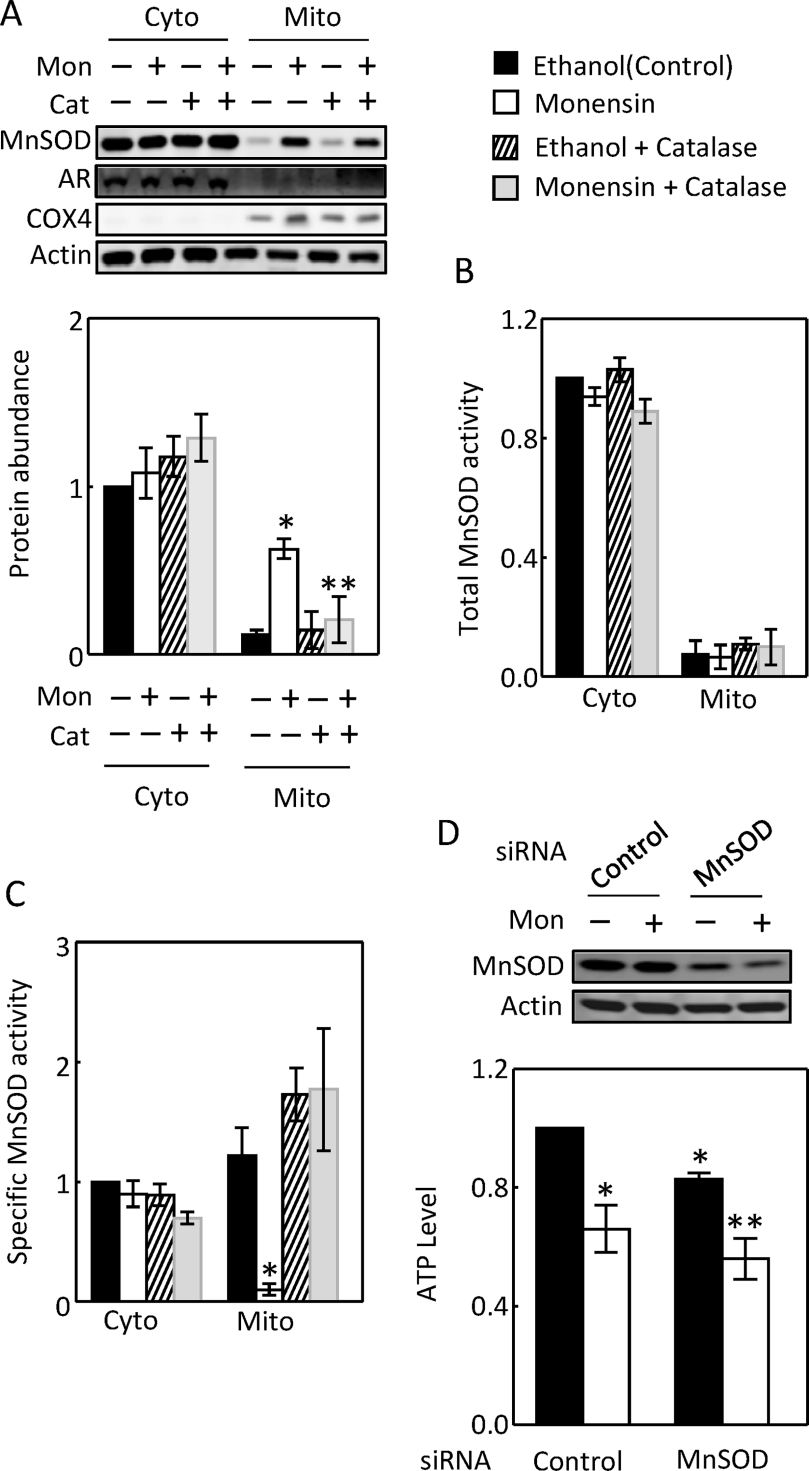
Catalase reduced the monensin-induced increase of mitochondrial MnSOD protein abundance (A, n = 5). The same membrane used in Fig 9A was incubated with the antibodies against MnSOD, AR, COX4 and actin (A). Catalase had no significant effect on the total MnSOD activity (B, n = 5), but reversed the monensin-induced inhibition of the specific MnSOD activity (C, n = 5). Knockdown of MnSOD reduced cellular ATP level with or without monensin (D, n = 4). The protein levels, total and specific activities of MnSOD were measured as in Fig 7. HEK293 cells were transfected with 80 nM control or MnSOD siRNA overnight and treated with or without monensin for 24 hours. The cellular ATP levels were measured as in Fig 5. (*p < 0.05 vs Control or Monensin plus Catalase in C, **p < 0.05 vs Monensin in A and control siRNA plus ethanol in D, Two-way ANOVA).

Catalase (400 U/ml) abrogated the effect of monensin on the mitochondrial MnSOD protein abundance (Fig 11A), indicating that the effect of monensin was mediated by ROS. Similarly, monensin had no significant effect on the total activity of either cytosolic or mitochondrial MnSOD, but reduced the mitochondrial specific MnSOD activity (Fig 11B & 11C). Catalase inhibited the effect (Fig 11B & 11C). To determine whether MnSOD is critical for the mitochondrial function, we used RNA interference to knock down MnSOD. We found that the knockdown of MnSOD modestly but significantly reduced the cellular ATP level in the absence or presence of monensin, indicating that MnSOD is important for mitochondria to generate ATP (Fig 11D). The transfection of control siRNA caused monensin to reduce the cellular ATP level, probably due to the harmful effect of transfection itself (Fig 11D).

## Discussion

### EAE increases mitochondrial function in the kidney cortex

MS and EAE primarily affect the immune and central nervous systems, but this relatively localized effect does not rule out the possible contributions by other organs to the pathogenesis of the diseases. For example, MS patients have few gastrointestinal complications, but the gut microbiota have been increasingly recognized as an important player in pathogenesis of the disease [1]. Although there have been few reports concerning metabolic-related kidney damage in MS and EAE, the kidney could contribute to pathogenesis of the diseases by increasing Na reabsorption. Our previous studies demonstrate that progression of EAE up-regulates the major Na transporters such as NHE3 and Na,K-ATPase in the kidney cortex [12]. In present studies we examined the energy generation necessary for the Na,K-ATPase to function. We show that EAE increased the mitochondrial complex II and IV activities, but not the complex I activity. These effects came with increases of the mitochondrial ability to generate ROS, mitochondrial MnSOD protein abundance and activity, and decrease of the specific activity of mitochondrial MnSOD (Figs 1 to 4).

### The activation of Na,K-ATPase by EAE in the kidney cortex may explain the effect of EAE on mitochondrial function

The effects of EAE on the mitochondrial function and MnSOD could be secondary to its effect on the Na transporters or resulted from inflammation independent of its effect on the Na transporters. For example, proteinuria is associated with MOG_92-106_-induced EAE [36], although whether this is the case in MOG_35-55_-induced EAE is unknown. Proteinuria increases mitochondrial function in the renal cortex [37]. Further, EAE increases serum interferon-gamma level [38], which is known to induce MnSOD [39]. The mechanisms by which monensin stimulates Na transport are similar to the ones for NHE3-mediated Na absorption in the kidney proximal tubules. To explore whether activation of Na,K-ATPase by EAE could be a mechanism underlying the effect of EAE on the mitochondrial function in the kidney cortex, we examined the effects of monensin in HEK293. We show that monensin simulated the effects of EAE on the complex II activity, mitochondrial ability to generate ROS, and mitochondrial MnSOD protein abundance and specific activity (Figs 5–11). Consistent with our observations is that monensin at low doses also increases mitochondrial activity in bovine spermatozoa, although which complex activity was not examined [40]. Both ouabain and catalase inhibited these effects (Figs 5 to 11). Since catalase also inhibited monensin-induced increase of Na,K-ATPase activity (Fig 9), these two mechanistically different agents provide strong evidence illustrating that activation of Na,K-ATPase is the mechanism accounting for the effects of monensin. These data suggest that the effects of EAE progression on the mitochondrial activity, ability to generate ROS and MnSOD in the kidney cortex are most likely derived from stimulation of Na,K-ATPase activity in the region. It is also possible that catalase attenuated the effects of monensin on mitochondrial ROS and MnSOD by direct decomposition of hydrogen peroxide. The significance of these effects in the pathogenesis of EAE is currently under investigation. Our results suggest that ouabain at 4 nM inhibits Na,K-ATPase activity in HEK293 cells (Figs 5 to 8). It is noteworthy that ouabain stimulates Na,K-ATPase at nanomolar ranges in murine tissues and cells [41, 42], whereas the concentration of ouabain has to be reduced below nanomolar in HK2 cells, a human kidney cell line, to produce a similar effect [42]. The reason is that Na,K-ATPase from different species has different sensitivity to ouabain [43].

It has been known that the acute stimulation of Na,K-ATPase by monensin increases mitochondrial respiration as measured by oxygen consumption [17] and mitochondrial motility [44]. Monensin also acutely decreases cellular ATP levels due to the increase of ATP consumption by Na,K-ATPase [45, 46]. However, the effect of long term monensin treatment on the mitochondrial activity is unknown. The present studies demonstrate that the monensin-induced activation of Na,K-ATPase for 24 hours specifically increased the mitochondrial complex II activity, not complex I or complex IV activity (Figs 5 and 10).

In contrast to the autoimmune disease systemic lupus erythematosus whose renal complications have been well documented [47], there have been only a few studies concerning the effects of MS on the kidney function. The renal function in MS has been generally considered normal and the observed renal damages are ascribed to either renal toxicity of medications or bladder dysfunction [36]. However, studies have shown that MS patients could have impaired glomerular filtration rate (GFR) not attributed to medications [48]. Myelin antibody deposits in the kidney, glomerular hypercellularity and proteinuria were also found in MOG_92-106_-induced EAE model [36]. Here we demonstrate that EAE progression increased mitochondrial ROS and MnSOD. Whether these increases affected renal function remains to be determined.

### MnSOD is present in the cytosol of the kidney and HEK293 cells and active there

MnSOD has been long regarded as being typically restricted in the mitochondrial matrix. In 2005, Luk et at reported that MnSOD is localized in both the cytosol and mitochondria in *Saccharomyces cerevisiae* [49]. However, the cytosolic MnSOD is largely inactive due to the absence of Mn. The mitochondrial MnSOD gains activity when the newly synthesized apo enzyme acquires Mn during its import to the mitochondria [49]. In 2010, Li et al documented for the first time that MnSOD is also present in the cytosol of mammalian cells by showing its presence in the cytosol of the human esophageal cell line, Het-1A [31]. The present studies demonstrate that MnSOD is present in the cytosol of both the kidney cortex (Fig 4) and HEK293 cells, and it is active there (Figs 4, 7 and 11). Surprisingly, a majority of the MnSOD protein abundance and activity are present in the cytosol of HEK293 cells (Figs 7 and 11). The abundant cytosolic MnSOD masked the effects of monensin on the mitochondrial MnSOD, when the total cellular extracts were used (data not shown). However, in contrast to the mitochondrial MnSOD, neither the cytosolic MnSOD protein abundance nor its activity is regulated by monensin, indicating that regulatory mechanisms for MnSOD in these two compartments differ. Further, the specific role of the cytosolic MnSOD remains to be defined.

### A possible mechanism for the effect of EAE on MnSOD in the kidney cortex

Mitochondrial superoxide acts as a signaling molecule at the physiological level [16], however, it becomes detrimental when it is produced beyond the control of MnSOD [50]. Therefore, MnSOD plays a critical role in the mitochondrial signaling pathway. However, MnSOD is vulnerable to inactivation by ROS, especially by peroxinitrate, which nitrates its tyrosine residues [51]. This is because mitochondria are a primary locus for the intracellular formation of peroxinitrate from superoxide and nitric oxide [52]. This knowledge may explain our observations in HEK293 cells. Monensin increased the mitochondrial ROS (Figs 6 and 8) and decreased the specific activity of mitochondrial MnSOD (Figs 7D and 11C). Ouabain and catalase reversed the effects of monensin (Figs 6, 8, 7D and 11C), indicating that the effect of monensin on the specific activity of mitochondrial MnSOD is mediated by the mitochondrial ROS. In order to protect the monensin-induced mitochondrial ROS from reaching a toxic level, monensin increased the mitochondrial MnSOD protein abundance to compensate for the ROS-induced loss of MnSOD-specific activity (Figs 7 and 11). However, monensin did not increase the total MnSOD activity in order to leave the mitochondrial ROS above the control level to mediate the effects of monensin on Na,K-ATPase and MnSOD (Figs 7, 9 and 11). It is speculated that severe EAE may use a similar mechanism to decrease the specific activity of mitochondrial MnSOD and increase the total mitochondrial MnSOD protein abundance and activity.

### Disclaimer

The content and views expressed in this article are the sole responsibility of the authors and do not necessarily reflect the views or policies of the Department of Defense or US Government. Mention of trade names, commercial products, or organizations does not imply endorsement by the Department of Defense or U.S. Government.

## Abbreviations

AR: aldose reductase
COX4: cytochrome c oxidase subunit IV
EAE: experimental autoimmune encephalomyelitis
MnSOD: manganese superoxide dismutase
MS: multiple sclerosis
NHE3: Na-H exchanger 3
